# Molecular Imaging Advances in Endometriosis: The Promise of Radiopharmaceuticals

**DOI:** 10.3390/molecules31010093

**Published:** 2025-12-25

**Authors:** Rebecca Napolitano, Giorgia Speltri, Petra Martini, Francesca Porto, Lorenza Marvelli, Alessandro Niorettini, Licia Uccelli, Luca Urso, Luca Filippi, Hatice Uslu, Burak Canitez, Hamza Alperen Kösem, Alessandra Boschi

**Affiliations:** 1 Department of Translational Medicine, University of Ferrara, Via Luigi Borsari 46, 44121 Ferrara, Italy; rebecca.napolitano@unife.it (R.N.); francesca.porto@unife.it (F.P.); licia.uccelli@unife.it (L.U.); luca.urso@unife.it (L.U.); 2 Department of Chemical, Pharmaceutical and Agricultural Sciences, University of Ferrara, Via Luigi Borsari 46, 44121 Ferrara, Italy; giorgia.speltri@unife.it (G.S.); lorenza.marvelli@unife.it (L.M.); 3 Department of Environmental and Prevention Sciences, University of Ferrara, Via Luigi Borsari 46, 44121 Ferrara, Italy; petra.martini@unife.it (P.M.); alessandro.niorettini@unife.it (A.N.); 4 Department of Biomedicine and Prevention, University of Rome “Tor Vergata”, Via Montpellier 1, 00133 Rome, Italy; luca.filippi@uniroma2.it; 5 Department of Nuclear Medicine, Istanbul Goztepe Prof. Dr. Suleyman Yalcin City Hospital, Fahrettin Kerim Gökay 161, Kadıköy, 34722 İstanbul, Turkey; uslusinav@hotmail.com (H.U.); burak.canitez@gmail.com (B.C.); halperen52@gmail.com (H.A.K.)

**Keywords:** endometriosis, imaging, radiopharmaceuticals

## Abstract

Endometriosis is a highly prevalent, chronic gynecological disorder characterized by the ectopic presence of endometrial-like tissue, driving significant morbidity and chronic pelvic pain. Pathologically, it is increasingly recognized as a fibro-inflammatory condition involving extensive tissue remodeling and fibrosis. Current conventional imaging modalities, including ultrasound and MRI, are primarily morphological, while standard molecular imaging using Positron Emission Tomography (PET) tracers has shown limited diagnostic utility. [^18^F]Fluorodeoxyglucose (FDG) suffers from high physiological uptake in pelvic organs and inconsistent detection of lesions. Receptor-based tracers like [^68^Ga]Ga-DOTATATE have demonstrated uncertain efficacy. In contrast, radiopharmaceuticals targeting the Fibroblast Activation Protein (FAP) offer a promising molecular approach. FAP is specifically overexpressed by activated fibroblasts present in the stroma of endometriotic lesions, correlating significantly with tissue fibrosis (collagen content) and local immune infiltration (e.g., CD68 macrophages). This comprehensive review analyzes the landscape of radiopharmaceuticals for endometriosis imaging, contrasting the specific limitations of traditional metabolic and receptor agents with the molecular rationale and emerging evidence supporting the use of FAP Inhibitors (FAPI), positioning them as crucial, non-invasive tools for the future diagnosis and management of this challenging disease.

## 1. Introduction

Endometriosis is a chronic, steroid hormone-dependent disorder defined as the presence of ectopic functional endometrial-like tissue outside the uterine cavity. Endometriosis affects approximately 10% of women of reproductive age worldwide [1,2]. This prevalence translates into a profound global public health burden, characterized by significant morbidity, including chronic pelvic pain, dysmenorrhea, dyspareunia, and infertility. Epidemiological data indicate that 31–42% of patients presenting with chronic pelvic pain or infertility suffer from this condition [3]. Despite its clinical prevalence and severe impact on the quality of life, the precise etiology remains elusive, and the disease often presents with heterogeneous and non-specific symptoms, contributing to a substantial diagnostic delay [2]. On average, the time from symptom onset to a definitive diagnosis span approximately 6 to 7 years, underscoring the critical demand for timely, accurate, and non-invasive diagnostic approaches.

Currently, the definitive diagnosis of endometriosis mainly relies on surgical visualization of the pelvis, typically via laparoscopy, coupled with histological confirmation of the excised lesions. While this remains the diagnostic gold standard, its invasive nature and associated morbidity preclude its use for regular monitoring of disease progression or evaluation of therapeutic response. Moreover, while most endometriosis foci arise from the pelvic region (particularly within gynecologic organs such as the perimetrium, ovaries and fallopian tubes), some patients may present with a more aggressive form of disease, known as deep infiltrating endometriosis (DIE). DIE can involve other pelvic organs, including the rectum (and the bowel more broadly) and the bladder, but might also extend beyond the pelvis, affecting the thoracic cavity [4]. Therefore, accurate imaging plays a crucial role in the diagnosis and assessment of disease extent in patients with endometriosis. Conventional non-invasive imaging modalities possess significant limitations: ultrasonography (US) is primarily useful only for ovarian endometriosis (endometriomas), and Magnetic Resonance Imaging (MRI)—while useful for depicting soft tissue changes in the pelvis—is not effective in detecting extraovarian endometrial adhesions and intraperitoneal implants [5]. Therefore, a normal US, Computed Tomography (CT), or MRI scan cannot reliably exclude the possibility of endometriosis. The inability of current modalities to quantify key pathological hallmarks or detect extrapelvic lesions highlights a critical unmet clinical need.

To overcome these constraints, modern molecular imaging, particularly using Positron Emission Tomography (PET) or Single-Photon Emission Computed Tomography (SPECT), has emerged as a promising avenue. Unlike morphological imaging, nuclear medicine tracers target specific biological processes intrinsic to the disease pathophysiology, which includes chronic inflammation, angiogenesis, hormone dependence and, crucially, fibrosis.

PET and SPECT involve the use of intravenously administered radioactive substances, known as radiotracers (or radiopharmaceuticals), that are designed to accumulate in tissues exhibiting disease-related features, such as increased metabolic activity or the expression of specific biomarkers. Combining PET or SPECT with Computed Tomography (CT) into hybrid PET/SPECT-CT scanners provides both biological and anatomical data, enhancing the specificity and localization of findings. The development of total body PET/SPECT-CT scanners further improves resolution, quickens scans, and results in considerably lower radiation exposure—a factor particularly relevant for women of reproductive age [6]. A major clinical advantage of nuclear imaging is its non-invasive nature, which contrasts with diagnostic laparoscopy and allows for repeatable examinations. This feature is particularly relevant for a chronic and progressive disease such as endometriosis, enabling longitudinal monitoring of disease activity and objective evaluation of therapeutic response over time, thus supporting more personalized patient management. Furthermore, SPECT and PET-based techniques can provide whole-body imaging, offering simultaneous information on the location, extent, and functional activity of endometriotic implants. This capability is crucial given the heterogeneous and often disseminated nature of the disease, including deep infiltrating and extrapelvic manifestations (e.g., pulmonary, pleural, abdominal wall, or umbilical involvement), which are frequently missed or underestimated by conventional morphological imaging.

This review examines the current landscape of radiopharmaceuticals for endometriosis imaging, comparing the specific limitations of conventional metabolic and receptor-based agents with the molecular rationale and emerging evidence supporting the use of FAP inhibitors (FAPI), thereby positioning them as a promising and potentially pivotal tool in this field.

## 2. Conventional and Receptor-Targeted Radiopharmaceuticals

Historically, molecular imaging for endometriosis has been hampered by non-specificity and the high physiological background activity inherent in the female pelvis. Several tracers originally developed for oncological staging have been incidentally studied or evaluated in pilot trials for endometriosis, yielding generally disappointing results.

### 2.1. [^18^F]Fluorodeoxyglucose ([^18^F]FDG) PET/CT

2-deoxy-2-[^18^F]fluoro-D-glucose ([^18^F]FDG) is the most widely used PET tracer in oncology as it exploits the principle of increased glucose metabolism characteristic of malignant tissues, a phenomenon commonly referred to as the Warburg effect [7,8]. After intravenous administration, [^18^F]FDG enters cells through glucose transporter, primarily GLUT-1 and GLUT-3, which are frequently overexpressed in tumor cells [9]. Once inside the cell, the tracer is phosphorylated by hexokinase to FDG-6-phosphate; however, unlike glucose, it cannot undergo further metabolic processing due to the replacement of the hydroxyl group at position 2 with the radioactive isotope ^18^F. This results in metabolic trapping, meaning that the molecule becomes intracellularly sequestered, leading to an accumulation proportional to the rate of glycolysis and hexokinase activity [10]. This biochemical behavior forms the basis of [^18^F]FDG’s use as a marker of hypermetabolism, making it a sensitive tool for identifying cells with increased bioenergetic activity, such as neoplastic cells and, to a variable extent, those involved in inflammatory processes. Given that endometriosis is characterized by altered cellular activity and chronic inflammation, incidental uptake of [^18^F]FDG in endometriotic lesions has been reported in several case studies [2]. However, the diagnostic utility of [^18^F]FDG PET in endometriosis remains severely limited due to its inconsistency and lack of specificity [11]. [^18^F]FDG accumulates in tissues with elevated glucose metabolism, a feature shared not only by malignant but also by inflammatory conditions. Consequently, benign endometriotic lesions mimicking malignancy on [^18^F]FDG PET/CT have been repeatedly documented [12,13,14]. Fox et al. [2] in recent systematic review confirmed that [^18^F]FDG PET does not reliably identify endometriotic lesions, with per-lesion correlations between PET avidity and histologically confirmed endometriosis ranging from 0% to 55%, and per-patient positivity rates ranging from 0% to 82%.

Physiological pitfalls in the pelvis further complicate interpretation, as [^18^F]FDG PET/CT is subject to high physiological uptake in hormone-sensitive organs, a limitation particularly relevant in the gynecological setting [15]. Physiological accumulation in the endometrium and ovaries, especially in premenopausal women and during specific phases of the menstrual cycle, can significantly impair accurate evaluation of primary pelvic tumors [16,17]. Moreover, the maximum standardized uptake value (SUVmax) reported for [^18^F]FDG avid endometriotic lesions (approximately 1.8–5.6) are generally lower than those expected for most malignancies, yet they overlap with the range observed in other benign and malignant peritoneal conditions [18]. In conclusion, [^18^F]FDG PET is not routinely recommended for diagnosing endometriosis due to its low sensitivity and specificity.

### 2.2. ^99m^Tc-Labeled Red Blood Cells (^99m^Tc-RBC)

Technetium-99m (^99m^Tc)-labeled radiopharmaceuticals, visualized using Single-Photon Emission Computed Tomography (SPECT), represent a valuable alternative to positron emission tomography (PET) tracers due to the lower cost and wider availability of SPECT technology. The in vivo labeling of red blood cells with ^99m^Tc-pertechnetate is a simple procedure that is widely used in nuclear medicine studies for the evaluation of the blood pool [19]. The technique is based on the so-called “pretinning” phase, during which a small amount of stannous pyrophosphate is administered intravenously. The stannous ions (Sn^2+^) thus introduced gradually enter the erythrocytes and create the conditions necessary for the radionuclide to bind stably within the cells [20]. After an interval of approximately 20–30 min, sufficient to ensure adequate intracellular availability of Sn^2+^, ^99m^Tc-pertechnetate is injected. The pertechnetate rapidly diffuses into the erythrocytes, where it is reduced by the stannous ions already present. This reduction process facilitates the binding of technetium to intracellular components, particularly hemoglobin, resulting in a stable and homogeneous labeling of circulating red blood cells. The method has the advantage of not requiring any external manipulation of the blood, making it faster, simpler, and more easily reproducible compared to in vitro procedures [19]. The use of ^99m^Tc-labeled red blood cell (^99m^Tc-RBC) scintigraphy has been explored as an innovative, non-invasive approach to detect peritoneal endometriosis [21]. This strategy builds on the understanding that cyclical and spontaneous hemorrhage is a hallmark of the disease and may therefore serve as an imaging biomarker of active lesions.

By labeling autologous red blood cells with ^99m^Tc, this radiopharmaceutical enables visualization of hemorrhagic activity within endometrial implants. The likelihood of detection appears to correlate with lesion size and vascularization, as larger or more active implants (Grades 3–4) are typically associated with stronger radionuclide signals indicative of enhanced neovascularization. Preclinical investigations in rat models have demonstrated promising results. Most endometrial implants were successfully visualized, with focal hyperactive regions identified in approximately 73% of cases, sometimes as early as four hours post-injection. Quantitatively, the mean lesion-to-background ratio (2.015 ± 0.619) exceeded that obtained with ^131^I-labeled tamoxifen, suggesting improved image contrast and diagnostic sensitivity.

The method is technically straightforward, allows early image acquisition, and entails a relatively low radiation burden. Unlike biochemical markers, it provides direct spatial information on lesion localization and extent. However, some limitations remain. Approximately one-quarter of lesions escape detection, likely due to insufficient vascularization, the absence of microbleeding, or the presence of infection or adhesions. Moreover, the episodic nature of hemorrhage in endometriotic tissue introduces variability, which may account for false-negative results and limits the technique’s reproducibility in clinical settings.

### 2.3. Receptor Targeted Tracers: [^68^Ga]Ga-DOTATATE and [^18^F]FES

The clinical quest for reliable, non-invasive diagnostic tools for endometriosis has led researchers to explore receptor-targeted radiotracers, primarily focusing on [^68^Ga]DOTA-(Tyr3)-octreotate ([^68^Ga]Ga-DOTATATE) and 16α-[^18^F]fluoro-17β-estradiol ([^18^F]FES).

#### 2.3.1. [^68^Ga]Ga-DOTATATE

[^68^Ga]Ga-DOTATATE PET/CT utilizes a tracer designed to target somatostatin receptors, which are consistently overexpressed on the surface of neuroendocrine differentiated cells and B-lymphocytes. Somatostatin receptors (SST1, SST2, and SST5) have been reported to be expressed in human endometrial tissue, including ectopic lesions. Moreover, the eutopic endometrium of women with endometriosis has been shown to exhibit significantly higher expression levels of SST1, SST2, and SST5 compared with that of control women [22].

The production of the radiopharmaceutical begins with the radionuclide gallium-68, which is generated using a ^68^Ge/^68^Ga generator. The generator serves as a source of ^68^Ga, in which germanium-68, a radioactive isotope, is eluted to obtain ^68^Ga in the form of gallium chloride. The eluted gallium-68 is then used to label the peptide DOTATATE, a somatostatin analog that contains the chelator DOTA (1,4,7,10-tetraazacyclododecane-N,N′,N″,N‴-tetraacetic acid), which binds ^68^Ga in a stable manner. The labeling process is carried out under controlled pH and temperature conditions to ensure a stable complexation between ^68^Ga and DOTA. The reaction proceeds rapidly, with a high radiolabeling yield, and the final product, [^68^Ga]Ga-DOTATATE, is ready for clinical administration [23]. The utility of [^68^Ga]Ga-DOTATATE in endometriosis, however, remains uncertain based on current evidence. A single prospective pilot study investigating its use in suspected severe endometriosis found that [^68^Ga]Ga-DOTATATE PET/CT detected endometriosis in only 4 out of 12 participants (33%). This reflects a selective rather than ubiquitous expression of somatostatin receptors (SSTs) across different endometriotic lesion subtypes. Specifically, receptor expression was shown to be highly lesion-dependent. Pathological [^68^Ga]Ga-DOTATATE uptake was observed exclusively in patients with rectovaginal deep infiltrating endometriosis (DIE) and in one case of adenomyoma. Immunohistochemical analysis confirmed the presence of SST1, SST2, and SST5 in these lesions. In contrast, no pathological uptake was detected in cases of superficial peritoneal endometriosis or ovarian endometriomas, and immunohistochemistry confirmed the absence of SST1, SST2, and SST5 expression in these lesion types. Given that [^68^Ga]Ga-DOTATATE primarily targets SST2 and SST5, the lack of tracer uptake in peritoneal and ovarian lesions strongly suggests that these receptor subtypes are not expressed in those forms of the disease. While it showed higher sensitivity (57%) and specificity (80%) for rectovaginal deep infiltrating endometriosis (DIE), its overall utility remains uncertain, and it performed less accurately than MRI. It is also unclear whether the tracer uptake originates specifically within endometrial cells or the associated lymphocytic infiltrate [24].

#### 2.3.2. [^18^F]FES

[^18^F]FES is a synthetic estradiol analog labeled with the radioactive isotope fluorine-18, targeting the Estrogen Receptor alpha (ER alpha). It is produced via automated fluorination of an estradiol precursor at the C16 position using [^18^F] from a cyclotron, under strictly controlled conditions to ensure high radiochemical purity and preserved biological activity. [^18^F]FES binds selectively to estrogen receptors (ER), and the receptor-tracer complex can be internalized into cells, generating a strong signal at tumor sites. This enables in vivo visualization and quantification of ER-expressing lesions, supporting diagnosis, staging, and monitoring of response to hormonal therapy. As endometriosis is an estrogen-dependent disease whose lesions histologically resemble the endometrium, [^18^F]FES holds a strong biological plausibility for imaging the condition. [^18^F]FES is already established in oncology for characterizing estrogen-related tumors, including breast and uterine cancers [25]. The physiological uptake of [^18^F]FES in the endometrium is known to fluctuate depending on the phase of the menstrual cycle. While the overall diagnostic efficacy of [^18^F]FES PET/CT for endometriosis remains undetermined due to limited data, one small trial suggested highly promising results: [^18^F]FES PET/CT was reportedly able to discriminate scars and adhesions from active endometriotic lesions and correctly identified the presence or absence of endometriosis in 97.5% of cases [25]. Nevertheless, as this trial enrolled fewer than five participants, the evidence remains preliminary. Less promising results were obtained in a couple more experiences. In a study by George et al. [26] [^18^F]FES PET detected only one endometriosis lesion among the four patients examined. Oldan and colleagues [27] performed preoperative [^18^F]FES PET/MRI in 6 patients affected by suspected superficial or peritoneal endometriosis or extragenital DIE, using postoperative histopathology as the reference standard. MRI outperformed [^18^F]FES PET both in terms of per-patient sensitivity (67% vs. 50%) and per-lesion sensitivity (48% vs. 35%) and detected all lesion that showed increased [^18^F]FES uptake. The authors therefore concluded that [^18^F]FES PET/MRI provides no additional value over conventional pelvic MRI in the presurgical diagnosis of endometriosis.

While [^18^F]FES targeting of hormonal receptors constitutes a mechanistically robust strategy for the non-invasive detection of endometriosis, rigorous validation in larger, well characterized patient cohorts is essential to substantiate its diagnostic value [28]. Both receptor targeted tracers currently suffer from limited published data, constrained by small sample sizes, highlighting the ongoing need for extensive future investigation in this diagnostic field.

### 2.4. Integrin-Targeted Radiopharmaceuticals: ^99m^Tc-maraciclatide

**Figure 1:**
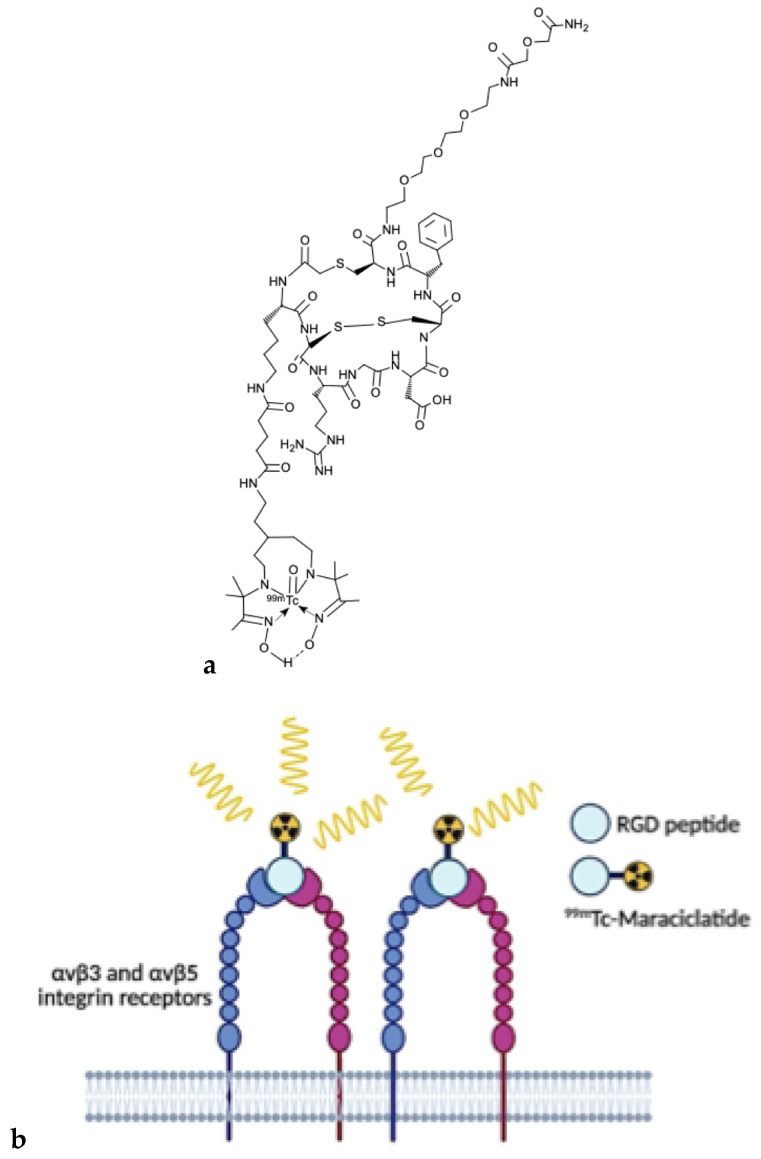
Chemical structure of ^99m^Tc-maraciclatide (**a**). Schematic representation of the binding of ^99m^Tc-maraciclatide to α_v_β_3_ and α_v_β_5_ integrin receptors (**b**).

^99m^Tc-maraciclatide (also known as [^99m^Tc-]-NC100692) is a cyclic peptide containing the Arg–Gly–Asp (RGD) motif, labeled with the radionuclide technetium-99m (Figure 1a). The peptide is designed to include a cyclic RGD site, which ensures high affinity and selectivity, and chelating groups (such as diamine dioxime) that enable stable binding to ^99m^Tc. The synthesis is performed starting from a lyophilized formulation, reconstituted with a solution containing the radionuclide of interest, to produce a radiolabeled peptide with high labeling yields and radiochemical purity suitable for clinical use. The mechanism of action of ^99m^Tc-maraciclatide is based on its interaction with α_v_β_3_ and α_v_β_5_ integrins, which are vitronectin receptors frequently overexpressed on proliferating endothelial cells, such as those involved in angiogenesis, as well as on other cell types, including osteoclasts [29] (Figure 1b).

These integrins are upregulated in pathological conditions characterized by angiogenesis and inflammation, including malignant breast tumors, rheumatoid arthritis, and bone metastases. The molecule incorporates a chelator that enables radiolabeling with the widely available ^99m^Tc, allowing visualization of tracer distribution and accumulation after intravenous administration using gamma scintigraphy or SPECT/CT.

The diagnostic potential of ^99m^Tc-maraciclatide has been investigated in both preclinical and clinical studies for the detection of bone metastases, malignant breast tumors, atherosclerotic plaques, rheumatoid arthritis, and peripheral vascular disease [30]. Notably, it has shown particular promise in identifying synovitis in rheumatoid arthritis, prompting further exploration in conditions with similar molecular and cellular features, such as endometriosis [31]. ^99m^Tc-maraciclatide has been demonstrated to be safe and well tolerated in healthy adult volunteers, with no serious adverse events (SAEs) reported. The radiopharmaceutical formulation has a dosimetry profile similar to other ^99m^Tc-based imaging agents used in nuclear imaging. The mean effective dose (ED) per unit injected activity was 7.8 ± 0.8 μSv/MBq. A major limitation is the biodistribution profile, which shows significant physiological uptake in organs located in or adjacent to the common sites of endometriosis. Initial uptake is highest in the gastrointestinal (GI) tract (20%) and liver (15%); the primary route of excretion is renal (55%); The highest absorbed dose occurred in the urinary bladder wall (36.2 μGy/MBq); This high activity in the GI tract, liver, and urinary system/bladder contents (which often fill the pelvis) could interfere with the optimal assessment and detection of small endometriotic lesions, especially those located on the peritoneal surfaces or in the deep pelvis [32].

In a recent study conducted by Gibbons et al. [33] preliminary data were presented from the first ten patients with known or suspected endometriosis. Each participant underwent SPECT/CT imaging using ^99m^Tc-maraciclatide, followed by planned laparoscopic surgery to verify the presence, extent, and anatomical distribution of endometriotic lesions. The study reported three key observations spanning the principal subtypes of endometriosis. ^99m^Tc-maraciclatide successfully identified superficial peritoneal endometriosis in patients later confirmed to have early-stage disease at laparoscopy. This form, accounting for nearly 80% of all endometriosis diagnoses, was the most frequently visualized, particularly along the thin peritoneal lining of the abdominal and pelvic cavities. Moreover, deep infiltrating endometriosis and endometriomas were also detected, with imaging findings showing strong concordance with laparoscopic verification.

Notably, the trial demonstrated a high correlation between the sites of maraciclatide uptake on SPECT-CT and laparoscopic findings across all endometriosis subtypes, including superficial peritoneal endometriosis, which remains challenging to visualize using current non-invasive imaging techniques.

These preliminary results suggest that α_v_β_3_-integrin–targeted molecular imaging could offer a promising route toward more accurate, non-invasive detection of active endometriotic disease. However, the implementation of an equivalent PET tracer would be highly desirable. PET imaging offers superior spatial resolution compared with SPECT, a feature that could be crucial for identifying very small lesions—an essential capability in what is often a true “treasure hunt” in patients with endometriosis. A PET-based α_v_β_3_-integrin tracer could therefore substantially enhance lesion detectability, improve diagnostic confidence, and potentially expand the clinical utility of molecular imaging in this complex disease.

## 3. FAPI Radiopharmaceuticals: Targeting the Fibro-Inflammatory Microenvironment

The greatest promise of molecular imaging in endometriosis lies in targeting the disease’s key pathological features, fibrosis and chronic inflammation. One of the most promising strategies involves imaging the Fibroblast Activation Protein (FAP), a well-established biomarker of activated fibroblasts within pathological stroma [5]. FAP is a type II serine protease that is minimally expressed in normal adult tissues but is markedly upregulated in cancer-associated fibroblasts (CAFs) across more than 90% of human epithelial carcinomas. Critically for endometriosis, FAP upregulation is also initiated by benign conditions such as inflammation, hormonal influence, and wound healing [34]. Studies carried out by Kellers and coworkers have confirmed the presence and significance of FAP expression in endometriotic lesions [3]. In an effort to elucidate the involvement of FAP in endometriosis, the authors examined 245 tissue specimens obtained from 159 patients, encompassing lesions from multiple anatomical locations. Using immunohistochemistry to visualize FAP-positive activated fibroblasts, they generated digital images for quantitative comparison. Strikingly, FAP expression was detected in 84% of endometriotic lesions, whereas only 4% of the distant control tissues showed any detectable signal. Across all analyzed sites, FAP levels were consistently and significantly elevated in endometriotic compared with non-lesional tissue.

Beyond confirming the presence of activated fibroblasts, the study provides insight into the microenvironmental context of endometriotic lesions. FAP expression correlated with both increased iron deposition—an indicator of lesion age—and enhanced collagen accumulation, suggesting a role in tissue remodeling and fibrosis. Furthermore, lesions rich in FAP-positive fibroblasts exhibited higher numbers of infiltrating immune cells, notably macrophages and CD8^+^ T lymphocytes, highlighting potential crosstalk between stromal activation and immune responses in endometriosis pathophysiology.

**Figure 2:**
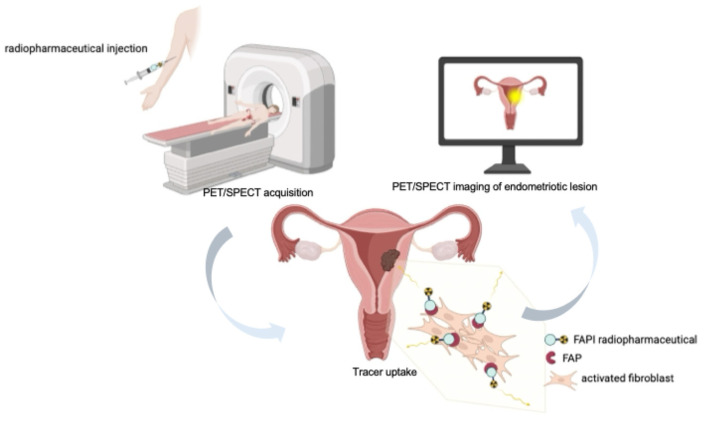
Schematic representation of FAPI-based PET or SPECT imaging of an endometriotic lesion. After intravenous injection of a FAP inhibitor (FAPI) radiopharmaceutical, the tracer circulates systemically and selectively binds to fibroblast activation protein (FAP) expressed on activated fibroblasts within the endometriotic lesion. The accumulated radioactivity is then detected by PET or SPECT imaging, enabling high-contrast visualization of FAP-expressing fibrotic and inflammatory tissue components.

Radiopharmaceuticals based on FAP inhibitors (FAPI) are small-molecule ligands (often quinoline-based) that bind with high affinity and selectivity to FAP [35]. When coupled to suitable radionuclides via bifunctional chelators (e.g., DOTA, NOTA, or HYNIC), these compounds enable both positron emission tomography (PET) and single photon emission computed tomography (SPECT) applications, as well as theranostic use when labeled with therapeutic radionuclides. After intravenous administration, FAPI tracers circulate and specifically bind to FAP expressed on activated fibroblasts in the tumor stroma or in fibrotic/inflammatory lesions. This leads to focal tracer accumulation at sites of disease (Figure 2). When labeled with diagnostic radionuclides (e.g., ^68^Ga, ^18^F, ^99m^Tc, or ^111^In), FAPI compounds provide high-resolution molecular imaging; when labeled with therapeutic radionuclides (e.g., ^177^Lu or ^90^Y), they can deliver targeted radiotherapy, integrating diagnostic and therapeutic capabilities in a single molecular platform.

### 3.1. FAPI-PET Tracers

The development of FAPI radiopharmaceuticals, such as [^68^Ga]Ga-FAPI-04 and [^18^F]AlF-NOTA-FAPI-04, capitalizes the specific targeting of this stromal protein.

In a recent preclinical study [5], an endometriosis model was successfully established in Sprague–Dawley rats through autologous transplantation of uterine endometrial tissue to the contralateral ovary and abdominal wall. Using small-animal PET/CT imaging performed 60 min after injection of [^18^F]AlF-NOTA-FAPI-04, the authors demonstrated markedly increased tracer uptake in ovarian endometriotic lesions (SUV_max_ = 2.53 ± 0.61) compared to both adjacent non-lesional ovarian tissue (1.28 ± 0.46, *p* = 0.031) and contralateral ovarian tissue (1.15 ± 0.49, *p* = 0.016). Similarly, peritoneal endometriotic lesions exhibited significantly higher uptake (SUV_max_ = 1.74 ± 0.15) relative to surrounding peritoneal tissue (0.97 ± 0.13, *p* = 0.001) and abdominal wall muscle (0.83 ± 0.12, *p* = 0.0004). This high contrast is crucial for overcoming the limitations faced by [^18^F]FDG imaging.

Histopathological and immunohistochemical analyses corroborated these imaging findings. Collectively, these results provide compelling evidence that [^18^F]AlF-NOTA-FAPI-04 PET/CT enables clear differentiation between endometriotic and normal tissues, underscoring its promise as a non-invasive imaging modality for the detection and characterization of endometriosis.

**Figure 3:**
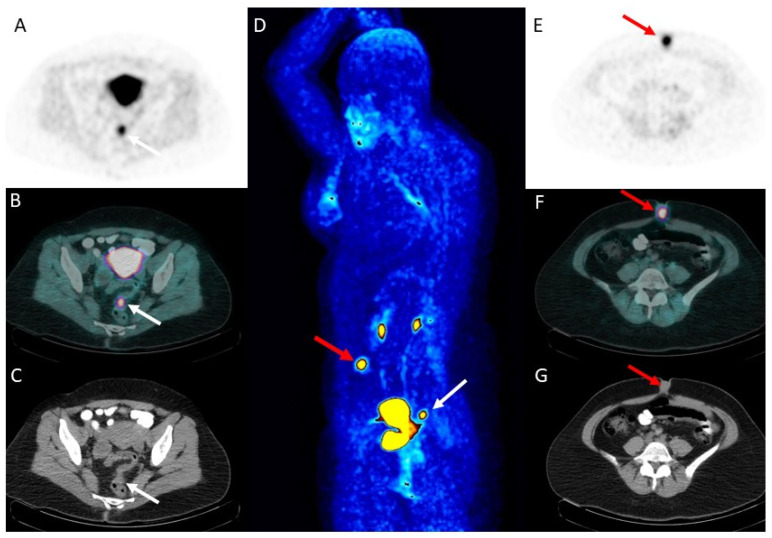
A 38-year-old female underwent [^68^Ga]Ga-FAPI-04 PET/CT due to suspected endometriosis. [^68^Ga]Ga-FAPI-04 PET/CT [(**A**,**E**): emission trans-axial PET images; (**B**,**F**): trans-axial fused PET/CT images; (**C**,**G**): trans-axial CT images; (**D**): maximum intensity projection—MIP] shows physiological radiotracer accumulation in the kidneys and bladder due to renal excretion and in correspondence with the uterus. Moreover, two areas of focal radiotracer uptake are detected in the rectum/sigmoid colon (white arrows) and in the umbilical area (red arrows). Following [^68^Ga]Ga-FAPI-04 PET/CT, the lesion detected at the umbilical area was biopsied and confirmed as a focus of endometriosis.

While large-scale clinical validation studies are ongoing (e.g., NCT06792318), preliminary case reports strongly support FAPI’s diagnostic potential in humans (Figure 3). The work reported by Burgard et al. [15] shows a notable case of a patient with DIE in the sigmoid colon confirmed the utility of [^68^Ga]Ga-FAPI-04 PET/CT. The scan showed intense tracer uptake (SUV_max_ 9.8) in the known lesion and, critically, identified additional sites of deep infiltrating endometriosis (in the ovary and ligamentum teres uteri) previously undetected by MRI (SUV_max_ up to 11.3, 7.3, and 5.4, respectively). This demonstrated the ability of FAPI to visualize non-malignant, fibro-inflammatory peritoneal disease [15].

In the related context of gynecological oncology, FAPI PET/CT has proven highly effective in detecting peritoneal metastases in ovarian cancer, enabling a potential theranostic approach [36]. Meta-analyses conducted by Florit et al. [37] show that [^68^Ga]Ga-FAPI-04 provided higher pooled sensitivity for detecting peritoneal metastases (97% vs. 70%) and nodal metastases (97% vs. 88%) compared to [^18^F]FDG in ovarian cancer. This capability to accurately map peritoneal disease, which is the primary manifestation site of endometriosis, underscores FAPI’s high relevance for this benign condition [37].

### 3.2. FAPI-SPECT Tracers 

**Figure 4:**
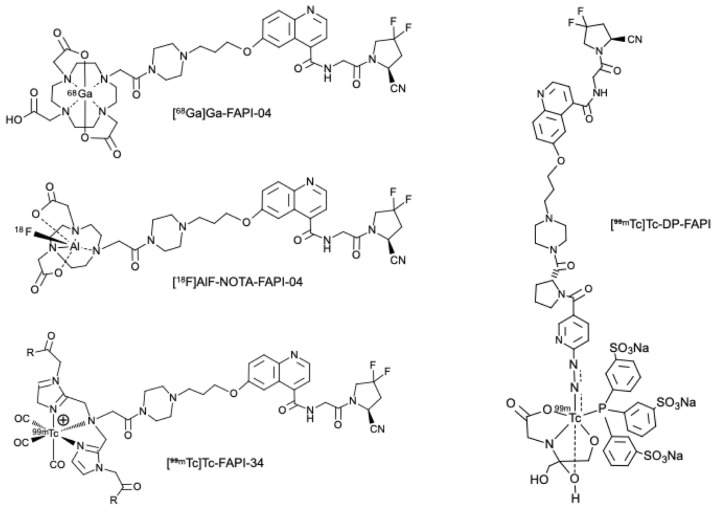
Chemical structures of FAPI radiopharmaceuticals.

Positron emission tomography combined with computed tomography (PET/CT) offers high diagnostic performance but remains limited by high costs and restricted accessibility compared to single-photon emission computed tomography (SPECT). To overcome these constraints, several fibroblast activation protein (FAP) inhibitors have been radiolabeled with technetium-99m (^99m^Tc), yielding SPECT tracers such as [^99m^Tc]Tc-FAPI-34, [^99m^Tc]Tc-DP-FAPI (Figure 4), and [^99m^Tc]Tc-iFAP [38]. These agents enable more affordable scintigraphic imaging while maintaining high diagnostic utility.

Current optimization strategies primarily focus on linker modification to improve pharmacokinetics—specifically, to minimize hepatobiliary excretion, which often limits image quality in ^99m^Tc-based tracers, and to enhance tumor accumulation. FAPI-34, for instance, was engineered to achieve high tumor-to-background contrast through rapid lesion uptake and prompt systemic clearance. The chelator used in FAPI-34 also allows labeling with therapeutic radionuclides such as rhenium-188 (^188^Re), whose half-life aligns well with the fast elimination kinetics typical of FAPI derivatives. Other analogs have been developed to accommodate isotopes like ^177^Lu and ^90^Y, enabling theranostic applications particularly relevant in oncology (e.g., soft-tissue sarcomas, pancreatic neoplasms) [38].

A notable advancement is the development of [^99m^Tc]Tc-DP-FAPI, synthesized through a straightforward kit-based procedure. This compound demonstrates strong affinity and selectivity for FAP in vitro and has shown excellent imaging performance in preliminary clinical evaluations. Early SPECT/CT studies in patients with various malignancies, including gastrointestinal cancers, confirmed its safety, high tumor contrast, and consistent lesion uptake. Moreover, promising diagnostic results have been reported in fibrotic diseases such as idiopathic pulmonary fibrosis and myocardial fibrosis, underscoring the tracer’s potential in non-oncologic indications.

Although large-scale clinical data are still lacking for endometriosis, the established overexpression of FAP in endometriotic tissue and encouraging results from preclinical PET/CT studies using [^18^F]AlF-NOTA-FAPI-04—capable of distinguishing ovarian from peritoneal lesions—suggest that ^99m^Tc-labeled FAPI tracers could represent a valuable tool for the noninvasive assessment of this condition in the near future.

### 3.3. Challenges: Physiological Uptake and Hormonal Influence in Gynecological Imaging

A major obstacle in applying FAPI imaging to the diagnosis of endometriosis, especially when compared with its use in non-pelvic malignancies, lies in the pronounced physiological uptake observed in female reproductive organs [1]. This uptake reflects not only inflammatory or reparative processes but also the strong influence of hormonal modulation on FAP expression. Diffuse [^68^Ga]Ga-FAPI-04 uptake is consistently observed throughout the uterus, particularly within the fundus and corpus of the myometrium, where SUV values can be roughly threefold higher than those measured in the cervix [16]. In a comparative analysis across cancer cohorts, endometrial FAPI uptake was markedly greater in premenopausal patients (SUV_max_ ≈ 11.7) than in postmenopausal ones (SUV_max_ ≈ 3.9), reflecting the dynamic, hormone-driven remodeling of the endometrium.

This hormonal dependence extends to cyclic variations: FAPI accumulation within the uterus and ovaries appears to fluctuate in concert with menstrual cycle phases. Uptake peaks in the uterus during the menstrual and proliferative phases, while ovarian activity rises around ovulation and into the early luteal phase.

Such pronounced physiological and hormonal modulation poses a significant interpretive challenge in FAPI PET/CT imaging of premenopausal patients. Elevated uterine uptake may obscure or mimic disease involvement, complicating the assessment of local extension and differentiation between pathologic lesions, such as endometriotic foci, and normal tissue remodeling. Similar interpretive difficulties may also arise in cervical cancer imaging, where hormonal variations further confound diagnostic accuracy.

To minimize these limitations, future clinical studies investigating FAPI imaging in endometriosis should carefully document and, where possible, standardize patients’ hormonal status, particularly the phase of the menstrual cycle. This will be essential to distinguish pathological FAP upregulation associated with fibrotic or inflammatory lesions from physiological changes driven by hormonal activity [5].

## 4. Comparative Performance and Future Perspectives

FAPI radiopharmaceuticals show intrinsic advantages over [^18^F]FDG due to lower background activity in most normal organs (e.g., brain, liver, pancreas), often resulting in superior contrast and diagnostic efficacy in detecting lesions (Table 1). This is confirmed in the broader gynecological oncology context, where FAPI tracers achieved comparable or superior detection rates and better TBRs than [^18^F]FDG for regional and distant metastases. Since endometriosis is fundamentally a fibro-inflammatory disorder defined by high FAP expression, FAPI PET/CT offers a non-invasive tool specifically adapted to visualize this underlying pathology. The ability of FAPI to accurately assess peritoneal disease severity, demonstrated in ovarian cancer, provides a strong indicator of its potential for mapping endometriosis.

To fully unlock the clinical potential of FAPI imaging in endometriosis, several key steps must be undertaken: (i) Large-scale validation: robust, prospective clinical trials are urgently needed to establish the sensitivity, specificity, and diagnostic accuracy of FAPI PET/CT across the full spectrum of endometriosis phenotypes, anatomical sites, and disease stages: (ii) standardized imaging protocols: harmonized acquisition protocols should be developed to minimize the confounding effects of hormonal fluctuations. This may involve carefully timing imaging sessions in relation to the menstrual cycle or the patient’s hormonal treatment status; (iii) molecular insights: further mechanistic studies are essential to elucidate the role of FAP in the fibrotic and inflammatory processes underlying endometriosis. Such insights could pave the way for novel FAP-targeted therapeutic strategies; (iv) multimodal integration: Combining FAPI PET/CT or PET/MR with high-resolution morphological imaging and image-guided biopsy techniques will be crucial to enhance diagnostic specificity—particularly in distinguishing endometriosis from inflammatory or neoplastic lesions that also demonstrate FAP expression.

In summary, FAP-targeted radiopharmaceuticals represent a promising, mechanism-driven innovation for the imaging of endometriosis. By visualizing the active fibro-inflammatory components of the disease, FAPI imaging has the potential to overcome the diagnostic variability and limitations associated with conventional tracers such as [^18^F]FDG, thereby transforming the non-invasive diagnostic approach for millions of affected women. Future research is needed to determine whether physiological uterine uptake truly limits the ability of this imaging modality to detect pelvic endometriosis foci (particularly those involving the perimetrium) or whether increased lesional uptake remains sufficient for detection despite the high background activity. Since endometriosis is characterized by pronounced neo angiogenesis and inflammation, processes also effectively targeted by ^99m^Tc-maraciclatide, this tracer may offer a valuable tool for diagnostic imaging. Current research is focused on evaluating integrin expression in endometriosis using ^99m^Tc-maraciclatide. From a safety perspective, ^99m^Tc-maraciclatide is well-tolerated, with a dosimetry profile comparable to other ^99m^Tc-based imaging agents, making it a feasible and accessible alternative to PET tracers.

Receptor imaging may represent a way to detect small endometriosis lesions. Despite extremely preliminary, current data published in the literature seem to suggest that [^18^F]FES PET may be more promising than [^68^Ga]Ga-DOTATATE PET. Nevertheless, the few papers currently available in the literature show extremely discrepant results: some indicate a very high accuracy [25], while others suggest that MRI might still outperform [^18^F]FES PET in endometriosis patients [27]. Further literature evidence with larger cohorts is needed to fully address the potential of [^18^F]FES PET in patients affected by endometriosis.

In addition to radiotracers already evaluated in clinical studies, several additional molecular targets with strong biological plausibility may be considered for the validation of existing probes or the development of novel radiotracers for endometriosis imaging. The chronic inflammatory nature of endometriosis, characterized by macrophage and mast cell infiltration, supports the exploration of targets involved in immune activation and cell recruitment, such as TSPO, COX-2, CCR2, and CXCR4. Imaging these pathways may provide functional insights into lesion activity that extend beyond purely anatomical assessment. Furthermore, targeting metabolic and hypoxia-related pathways—including amino acid transport, fatty acid metabolism, and hypoxia-inducible signaling—may complement FDG-based approaches by improving specificity and better reflecting the unique metabolic and microenvironmental features of endometriotic tissue. Collectively, these strategies highlight the potential of molecular imaging to further refine lesion characterization and to expand.

Although still at the embryonal phase, artificial intelligence (AI) models may enhance the diagnostic assessment of patients with endometriosis in the near future [39]. Some preliminary applications in this context have been reported with radiological imaging, mainly US and MRI. Albeit promising, a major limitation to training AI models in this context is the limited number of patients undergoing surgery, which restricts the availability of robust histopathological reference standards needed for accurate model development. To date, no AI models have been tested in endometriosis patients undergoing molecular imaging. However, future experiences in this context may guarantee to overcome some of the actual limitations of SPECT and PET imaging in patients affected by endometriosis.

In summary, molecular imaging, particularly using radiopharmaceuticals for PET, holds substantial promise to transform endometriosis care by enabling safe, non-invasive diagnostic tools and informing early treatment strategies. This advancement moves towards a more personalized and effective approach to managing this multisystem disorder. Further efforts are needed to advance our understanding of molecular imaging in endometriosis and to identify the most appropriate radiotracer for each specific clinical indication.

## Figures and Tables

**Table 1:** Comparative performance summary of radiopharmaceuticals for endometriosis imaging.

Radiopharmaceutical	Target	Efficacy in Endometriosis	Limitation
[^18^F]FDG	Glucose Metabolism	Inconsistent detection (0–55% per lesion) [2].	Lack of specificity; high physiological pelvic background [1].
[^68^Ga]Ga-DOTATATE	Somatostatin Receptor	Uncertain utility (33% detection rate) [24].	Low sensitivity; uptake source unclear [2].
^99m^Tc-maraciclatide	αvβ3 and αvβ5 Integrins	High biological plausibility (angiogenesis/inflammation) [32].	High Physiological Uptake in Pelvic/Abdominal Area. Limited Clinical Development Status [1,16].
[^68^Ga]Ga-FAPI-04	Activated Fibroblasts (FAP)	High lesion contrast; successful detection of DIE lesions (SUV_max_ up to 11.3) [5,15].	High physiological uptake in hormone-sensitive organs (uterus/breast) [1,16].

## Data Availability

No new data were created or analyzed in this study. Data sharing is not applicable to this article.

## Abbreviations

The following abbreviations are used in this manuscript:

PET	Positron Emission Tomography
FDG	[^18^F]Fluorodeoxyglucose
FAP	Fibroblast Activation Protein
DIE	deep infiltrating endometriosis
US	ultrasonography
MRI	Magnetic Resonance Imaging
SPECT	Single-Photon Emission Computed Tomography
CT	Computed Tomography
DOTA	1,4,7,10-tetraazacyclododecane-N,N’,N’’,N’’’-tetraacetic acid
SUV	Standardized Uptake Value
NOTA	1,4,7-triazanonane-1,4,7-triacetic acid
HYNIC	6-hydrazinonicotinic acid
ED	effective dose
GI	gastrointestinal
TBR	target background ratio
